# Phase Composition of Silica Fume—Portland Cement Systems Formed under Hydrothermal Curing Evaluated by FTIR, XRD, and TGA

**DOI:** 10.3390/ma14112786

**Published:** 2021-05-24

**Authors:** Eva Kuzielová, Michal Slaný, Matúš Žemlička, Jiří Másilko, Martin Tchingnabé Palou

**Affiliations:** 1 Institute of Construction and Architecture, Slovak Academy of Sciences, Dúbravská Cesta 9, SK-845 03 Bratislava, Slovakia; michal.slany@savba.sk (M.S.); matus.zemlicka@savba.sk (M.Ž.); martin.palou@savba.sk (M.T.P.); 2 Faculty of Chemical and Food Technology, Slovak University of Technology, Radlinského 9, SK-812 37 Bratislava, Slovakia; 3 Institute of Inorganic Chemistry, Slovak Academy of Sciences, Dúbravská Cesta 9, SK-845 36 Bratislava, Slovakia; 4 Materials Research Centre, Faculty of Chemistry, Brno University of Technology, Purkyňova 118, CZ-612 00 Brno, Czech Republic; masilko@fch.vut.cz

**Keywords:** hydrothermal curing, silica fume, FTIR

## Abstract

Two substitution levels of Portland cement by silica fume (SF; 30 and 50 mass%) and three hydrothermal treatment regimes (0.5, 1.2, and 2 MPa and 165, 195, and 220 °C for 7 days, respectively) were selected for the investigation of high-temperature phase formation. A combination of thermogravimetric, X-ray diffraction, and Fourier transform infrared analyses in the mid-IR region was used to overcome the shortcomings of individual techniques for the identification of these complex systems. Changes in molecular water amounts, the polymerization degree of silicate chains, or their decomposition due to transformations and crystallization of phases at hydrothermal conditions were observed and discussed concerning composition. Contrary to the calciochondrite, hydrogrossular phases, α-C_2_SH, and jaffeite detected in the systems without SF, a decrease in CaO/SiO_2_ ratio resulted in the formation of stable tobermorite in the case of 30 mass% SF, whilst calcium hydrogen silicate, gyrolite, and cowlesite were identified as more thermally stable phases in the samples with 50 mass% SF.

## 1. Introduction

The formation and stability of nanocrystalline and crystalline phases, which can be expected to occur in cementitious systems at higher temperatures and pressures for a significant time [1,2,3,4,5,6], attract attention of not only the practice, but also the scientific community. From the scientific point of view, it is important to establish phase diagrams or solubility models, which are necessary for the adjustment of cement mixtures compositions or formulation of concretes for special purposes [7]. The ultimate goal is, of course, to improve the durability of materials, which is influenced by different types of aggressions, including not only temperature and pressure but also the chemical aggressivity of geothermal water. Related findings are inevitable for applications, e.g., in the case of geothermal energy usage or for CO_2_ geological sequestration [8,9].

An equilibrium state under certain conditions is influenced by many factors. Among them, and taking Portland cement systems into account, the Ca/Si (C/S) ratio belongs to the most important ones. Whilst the Ca/Si ratio, which is present in the ordinary Portland cement pastes, varies from ≈1.2 to ≈2.3 with a mean of ≈1.75 [10], the decrease of C/S ratio with some silica source is a usual way how to partially prevent from transformations of primary hydration products at high temperatures and pressures, and thus to stabilize cement materials [11,12]. The lower the C/S ratio, the lower-base calcium silicate hydrates formation can be expected. A variety of possible structures exists, whereby decreasing calcium content results in increased silicate polymerization. According to the classification established by Strunz [13], which considers the silica chain polymerization, possible phases include a group of nesosubsilicates, sorosilicates, inosilicates, or phyllosilicates. The nesosubsilicates consist of isolated [SiO_4_]^4−^ groups and possess the lowest degree of polymerization. A typical example of this group occurring at high temperatures in the systems with a high C/S ratio is α-C_2_SH [14]. Jaffeite, often existing together with α-C_2_SH, belongs to sorosilicates, which are composed of [Si_2_O_7_]^6−^ groups connected to two isolated tetrahedra via one bridging oxygen to form the so-called pyrogroups. Inosilicates possess structures based on silicate chains (e.g., 11 Å tobermorite, xonotlite, hillebrandite) and phyllosilicates structures based on silicate sheets (e.g., Z-phase, truscottite, or gyrolite [15]).

Black et al. [16] detected increased hydroxide group binding energies with decreasing calcium content. In addition, significantly higher binding energies in the phyllosilicates than in the inosilicates were recognized as a result of their significantly greater Ca–O length. Dharmawardhana et al. [17] indicated that besides silicate polymerization [18,19], the order of bonds between each pair of atoms, representing bond stiffness and strength, also affects the deformation mechanisms of materials. Among different oxygen-bonding environments (such as bridging oxygen atoms adjoining adjacent silicate tetrahedral, non-bridging oxygen atoms connecting silicate tetrahedra with charge balancing calcium atoms, etc.), the role of hydrogen bonding should not be neglected, as it has an important role in the behavior of different cementitious structures [20,21,22].

Many analytical methods are used to investigate the structural features of crystalline or nanocrystalline C(-A)-S-H phases, such as X-ray diffraction (XRD) [23,24], Fourier transform infrared spectroscopy (FTIR) [25], ^29^Si magic angle spinning nuclear magnetic resonance spectroscopy (^29^Si MAS NMR) [26,27,28], or X-ray photoelectron spectroscopy (XPS) [24,29]. However, numbers of the corresponding studies are performed on the C-S-H phases synthesized from stoichiometric starting compositions or crystalline phases formed in nature. Contrary to them, real cementitious systems are much more complex, and as was highlighted by Blanc et al. [29], the influence of elements other than Ca or Si should be taken into account, as these could imply a displacement of the C-S-H solubility curve. These emphasize the need for detailed studying of composites systems, which already are or can be implemented in practice.

The present manuscript is a part of an extensive study focused on composite cementitious materials for deep hydrothermal well applications. Silica fume (SF) is a pozzolanic additive that is frequently mixed with the oil-well cement to prevent its degradation. Its effect on the pore structure and mechanical properties of cement submitted to different hydrothermal conditions is discussed in our former articles [30,31,32,33]. The present study poses advantages that result from the combination of thermogravimetric (TGA), XRD, and FTIR analyses, which allow detailed examination of different structures developed in the resulted materials.

## 2. Material and Methods

The composition of prepared samples, as depicted in Table 1, consists of CEM I 52.5 R (Považská cementáreň, a.s., Ladce, Slovakia) and silica fume (SF; Oravské ferozliatinárske závody, a.s., Slovak Republic). The oxide composition of input materials and mineral composition of the used cement are presented in Table 2 and Table 3, respectively.

Referential, as well as blended samples, were prepared by mixing dry mixtures with an adequate quantity of water (water-to-binder ratio (*w*/*b*) equal 0.44). In the case of the composite samples, plasticizer Stachement^®^ 903 (Stachema Bratislava, a.s., Rovinka, Slovakia) suitable for the given conditions was applied to reduce the water demand. The plasticizer concentration (0.5 g per 100 g of the binder) in water was kept constant. Appropriate homogenization was performed by laboratory cement mixer (MI-CM5, BETON SYSTEM, s.r.o., Brno, Czech Republic). Fresh cement pastes were poured into 16 × 4 × 4 cm molds and left to hydrate at 105 °C. To prevent drying, the samples were stored in a closed box above water. After 2.5 h, still hot samples were demolded, quickly put in laboratory autoclave, and exposed for 7 days to hydrothermal curing at 0.5, 1.2, and 2 MPa and 165, 195, and 220 °C, respectively (High Pressure Autoclave Testing Bluhm & Feuerherdt GmbH, Berlin, Germany).

Phase changes occurring in the samples were monitored by the TGA/DSC technique (TGA/DSC-1, STARe software 9.30, Mettler Toledo, Columbus, OH, USA). The 50.00 (±0.1) mg of powdered samples were heated in the open platinum crucibles for up to 1000 °C at the heating rate of 10 °C min^−1^ in the atmosphere of synthetic air (purity 5.0).

Crystalline phases in the samples were detected by XRD analysis (Diffractometer system EMPYREAN, PANanalytical, the Netherlands; CuKα radiation, λ = 0.1540598 nm, operating at 40 kV and 30 mA).

The infrared spectra were performed using a Nicolet 6700 FTIR spectrometer from Thermo Scientific™. The IR source, KBr beam splitter, and DTGS detector were used for the mid-IR (MIR) measurements (4000–400 cm^−1^). MIR transmission spectra were collected using the KBr pellet technique (1 mg of a sample homogenized with 200 mg KBr). KBr pellets were dried at 60 °C overnight. For each sample, 64 scans were recorded with a resolution of 4 cm^−1^. The Thermo Scientific package (OMNIC™ software) was used for the spectra manipulations and to detect the exact position of the vibrational bands appearing as inflections/shoulders in the IR spectra involving the Savitsky–Golay second derivatives.

### Structural Characterization of Raw and Modified Cement by FTIR Spectroscopy

Detailed examination and description of infrared spectra of cement materials, and especially hydrated multicomponent cement composites, is very difficult because of numerous phases that are present there, which result in the overlapping of their absorption bands. Therefore, the absorption bands are further discussed and assigned mostly related to the presence and structure of the main phases, or the phases with a significant impact on the properties.

**Figure 1:**
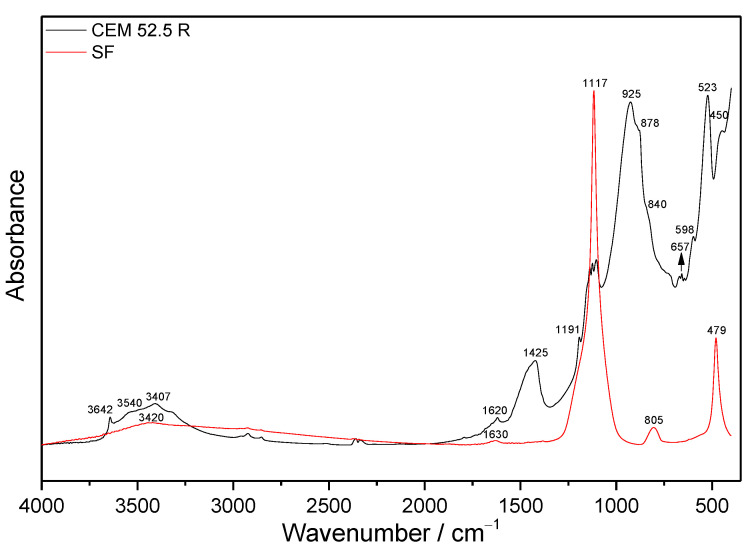
The MIR spectra of initial materials.

The infrared spectra of initial materials are displayed in Figure 1. The SF is composed mainly of amorphous SiO_2_, and its spectrum demonstrates characteristic Si-O vibrations of free SiO_4_ at about 1117, 805, and 479 cm^−1^. Only a negligible wide band centered close to 3420 cm^−1^ together with an absorption peak at 1630 cm^−1^ can be assigned to the bands of water molecules from SF and KBr [34].

The C_3_S, present as the main clinker phase in dry cement, demonstrates strong asymmetric (ν_as_) and symmetric (ν_s_) stretching vibration generated by Si-O bonds in [SiO_4_]^4−^ tetrahedral units at 925 and 878 cm^−1^, and symmetric (δ_s_) and antisymmetric (δ_as_) bending vibrations of O-Si-O at 523 and 450 cm^−1^, respectively. Strong stretching vibrations in silicate units of the second major calcium silicate clinker phase, C_2_S, exhibit only as a shoulder at about 840 cm^−1^ [35]. Other absorption bands overlap with those of C_3_S. Between wavenumbers 1180 and 1070 cm^−1^, the triple bands occur due to sulfate stretching vibrations, which probably suggests partial dehydration of gypsum. With decreasing wavenumber, the maxima are attributed to asymmetric and symmetric stretching vibrations in SO_4_^2−^ tetrahedra. The asymmetric bending vibrations coming from these groups in gypsum and bassanite are situated at 669, 598, and 657 cm^−1^, respectively [36,37]. Symmetric stretching vibrations of water in these sulfates show absorption peaks at about 3407 and 3540 cm^−1^, whilst particular antisymmetric stretching vibrations modes of water manifest at around 3489 cm^−1^. The sharp peak at 3642 cm^−1^ belonging to Ca-OH stretching vibrations in portlandite suggests partial hydration of cement. The bending vibration of water from cement and KBr is visible at 1620 cm^−1^. Partial carbonation of the cement during manipulation also manifests by the stretching vibrations in CO_3_^2−^ groups at about 1425 cm^−1^ [38].

## 3. Results and Discussion

### 3.1. FTIR Spectroscopy

**Figure 2:**
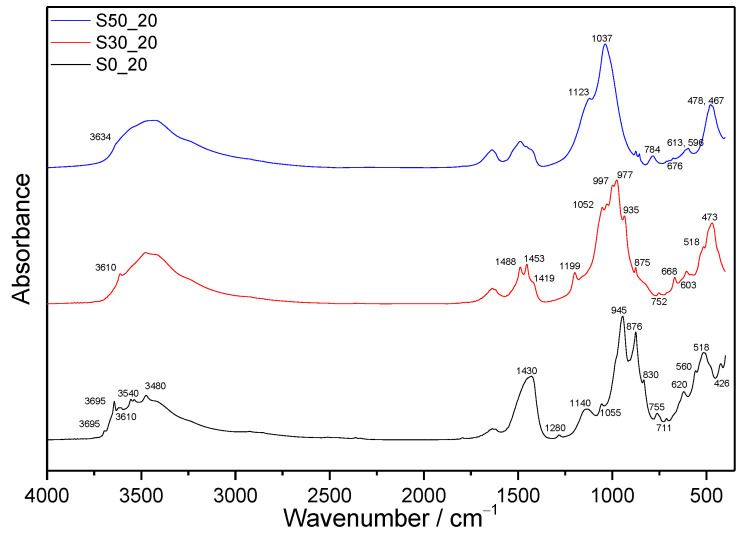
MIR spectra of samples hydrothermally treated at 2.0 MPa and 220 °C.

**Figure 3:**
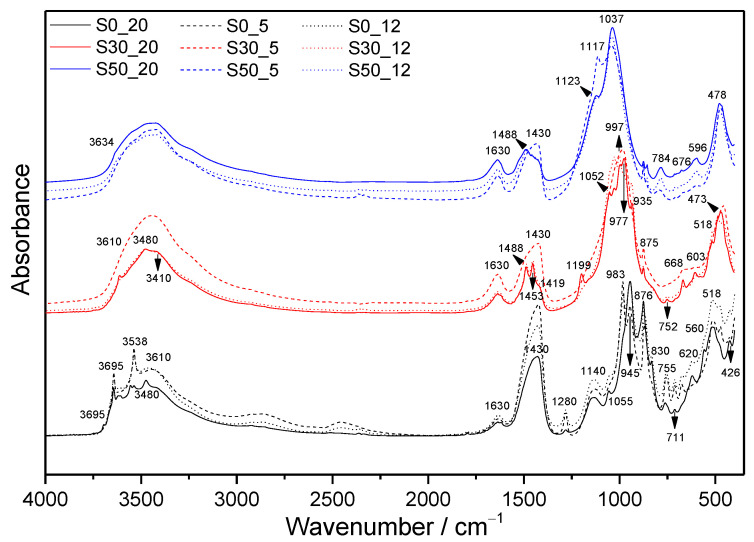
Comparison of MIR spectra of samples with different compositions and hydrothermal curing conditions.

**Figure 4:**
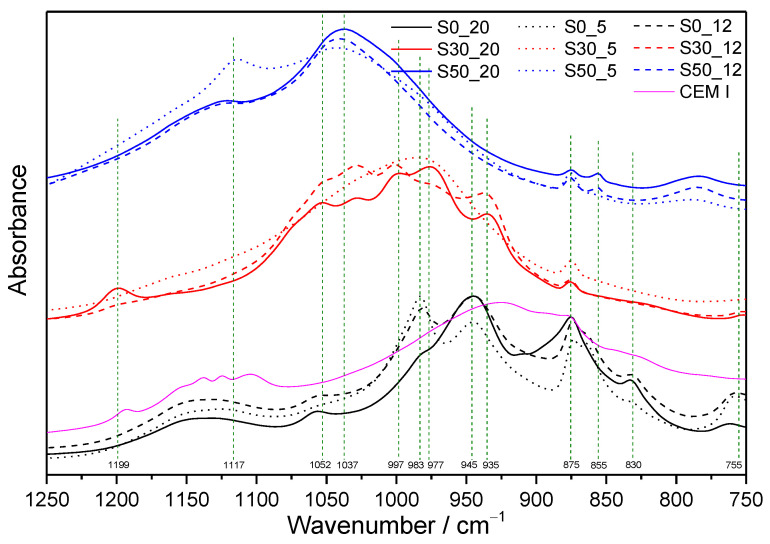
Segment of infrared spectra of all hydrated samples and referential dry cement between 1250 and 750 cm^−1^.

For the sake of clarity, whilst only the selected MIR spectra of the samples submitted to the more severe hydrothermal conditions (220 °C, 2 MPa) are depicted in Figure 2, the absorption spectra of all prepared and studied samples are collected in Figure 3. The segment of infrared spectra of all hydrated samples and referential dry cement between 1250 and 850 cm^−1^ is displayed in Figure 4.

#### 3.1.1. Samples without SF Addition

The first important mid-IR (MIR) region in the infrared spectra of hydrated cementitious materials includes absorption bands from water or OH species adsorbed to the surface, molecular water and absorption bands of a metal-bonded hydroxyl group. Contrary to the samples prepared with the addition of SF, the spectra of pure cement pastes contain a sharp peak due to stretching vibrations of Ca-OH from portlandite (≈3644 cm^−1^) [39]. The intensity of a particular band decreases with increasing temperature and pressure of autoclave treatment confirming the results from TGA (Figure 2).

**Figure 5:**
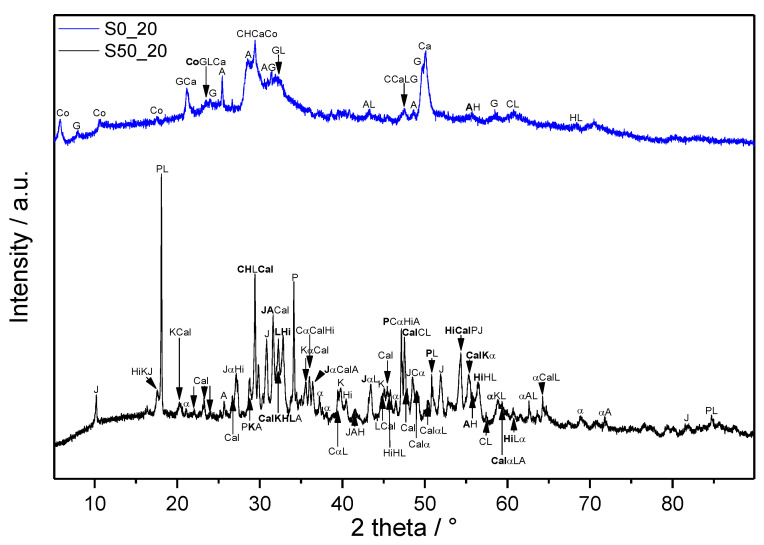
XRD patterns of samples cured at 2.0 MPa and 220 °C for 7 days. Abbreviations: α—α-dicalcium silicate hydrate (Ca_2_(SiO_3_OH)(OH), α-C_2_SH), A—anhydrite (CaSO_4_, $C\bar{S}$), C—calcite (CaCO_3_, C$\bar{C}$), Ca—calcium hydrogen silicate (CaH_4_Si_2_O_7_, CS_2_H_2_), Cal—calciochondrite (Ca_5_(SiO_4_)_2_(OH)_2_, C_5_S_2_H), Co—cowlesite (CaAl_2_Si_3_O_10_·6H_2_O, CAS_3_H_6_), G—gyrolite (Ca_8_Si_12_O_30_(OH)_4_·7H_2_O, C_8_S_12_H_9_), H—hatruite (Ca_3_SiO_5_, C_3_S), Hi—hibschite (Ca_3_Al_2_(SiO_4_)_3-x_(OH)_4x_, x is from 0.2 to 1.5), J—jaffeite (Ca_6_Si_2_O_7_(OH)_6_, C_6_S_2_H_3_), K—katoite (Ca_3_Al_2_(SiO_12_)_3-x_(OH)_4x_, x is from 1.5 to 3.0), L—larnite (Ca_2_SiO_4_, β-C_2_S), P—portlandite (Ca(OH)_2_, CH).

A small absorption peak at about 3695 cm^−1^ observed in the sample S0_20 is assigned to νAl-OH in hydrogrossular phases. Based on the results of XRD analyses (Figure 5), this stretching vibration originates from the structure of hibschite. Other stretching vibration bands of hibschite and the second present hydrogrossular, katoite, lying above 3400 cm^−1^, are partially overlapped with other absorption bands. The replacement of [SiO_4_]^4−^ tetrahedra by 4OH^−^, resulting in a range of intermediate compositions, has a strong influence especially on the bands in the region between 3620 and 3670 cm^−1^, and at approximately 620 cm^−1^, which is also presented in the MIR spectra. In the region of higher wavenumbers, hibschite shows two diffuse bands at 3660–3670 cm^−1^ and 3620–3630 cm^−1^, whilst katoite shows a complex band with a sharp and strong maximum at about 3650 cm^−1^. The later peak is contrary to the anhydrous grossular shifted toward lower wavenumbers in hibschite and absent in katoite [40].

In addition, Ca-OH stretching vibrations from crystalline jaffeite (≈3610 cm^−1^) and α-C_2_SH (≈3540 cm^−1^) can be observed in the spectra of S0. The presence of α-C_2_SH is confirmed also by characteristics peak at ≈755 cm^−1^ due to Si-OH stretching vibrations, and absorption peaks at ≈864 and 980 cm^−1^ owing to Si-O stretching vibrations in the SiO_4_ tetrahedron [25]. The latter two are visible only in the spectra of S0_5 and S0_12 treated at lower temperatures. The bands between 2320 and 3000 cm^−1^ are typical for OH stretching vibration when OH is involved in hydrogen bonding. The broadening of bands relating to hydrogen bonding is caused by their wide range of strengths, whereby the stronger hydrogen bonding, the lower the frequency of OH stretching vibrations is observed [41]. The strong Si-OH bending vibration of hydroxyl bonded to SiO_4_ (≈1280 cm^−1^) is visible in all S0 samples. At 675 and 711 cm^−1^, libration OH (Ca) from α-C_2_SH is observed [14]. Other typical absorption peaks of jaffeite can be found at 830 and 1055 cm^−1^ from Si-O and Si-O-Si stretching vibration, respectively [14]. Unlike absorption bands of α-C_2_SH, the intensity of bands belonging to jaffeite increases with temperature and pressure.

Diffuse bands, which can be observed at about 3450 and 1640 cm^−1^, are attributed to the stretching and bending vibrations of the physically adsorbed water molecules at the surface, respectively [42]. On the contrary, the sharp peak situated near 3480 cm^−1^, together with the absorption peak at 3550 cm^−1^ evident in the spectra of S0_20, indicates the presence of calciochondrodite [43]. In the region of lower wavenumbers, the bands of calciochondrodite are at about 945 and 876 cm^−1^. The absorption peak at approximately 765 cm^−1^ is partially overlapped with that of α-C_2_SH.

The presence of calcite and anhydrite is confirmed by the vibration modes between 1580 and 1080 cm^−1^. The peak at about 1430 cm^−1^ is caused by C=O stretching in CO_3_^2−^, whilst that at 1140 cm^−1^ is caused by S-O stretching in SO_4_^2−^.

In addition to the above-mentioned, the absorption spectra of S0_5 and S0_12 show still significant Si–O stretching vibration from C-S-H at about 980 cm^−1^ [44]. The intensity of Si-O-Si bending vibration at about 470 cm^−1^ also decreases with increasing pressure and temperature. A double peak attributed to Si-O-Al bending vibration appears around 518 cm^−1^. Unlike the previous ones, the intensity of peak at about 620 cm^−1^, attributed to hydrogarnets, increases with more severe conditions of autoclave treatment. However, possible overlapping with bending vibration from anhydrite in this position should be mentioned [35]. At about 560 cm^−1^ and 426 cm^−1^, Al-O stretching and bending vibration in octahedral AlO_6_ can be observed, respectively. The intensity of particular bands also increases with pressure and temperature.

#### 3.1.2. Samples with SF Addition

Additions of SF led to significant changes in the infrared spectra (Figure 2 and Figure 3). In the case of lower SF amount, the presence of jaffeite is confirmed only in the samples S30_12 and S30_20, whilst any fundamental bands belonging to this phase appear in the spectra of samples with higher SF addition. The characteristic bands of α-C_2_SH are not visible. However, they can be overlapped by more pronounced bands of other phases. The sharp peak of portlandite observed in the region of OH stretching vibrations disappeared already as a result of the lower amount of SF. In addition, only Ca-OH stretching vibrations from hydrogrossular are visible in the spectra of S30_12 and S30_20. The intensity of the diffuse band lying in the region between 3700 and 3100 cm^−1^ is higher than in the case of samples without SF additions, which confirms higher amounts of molecular water incorporated into the structure of formed phases. The same findings are concluded based on TGA (Section 3.3.2).

In the region of C=O asymmetric stretching vibrations in CO_3_^2−^, the samples submitted to higher pressures and temperatures show two absorption bands and a shoulder at about 1488, 1453, and 1419 cm^−1^, respectively [45]. The occurrence of several peaks suggests that besides calcite, other phase modifications of CaCO_3_ were formed under these conditions as well. On the contrary, in the case of samples S30_5 and S50_5, the splitted peaks merged and formed a broad band with a maximum near 1419 cm^−1^, which suggests that calcite is the dominating calcium carbonate. Neither aragonite, nor vaterite was identified using XRD; however, this can be explained by the lower detection sensitivity of this method in comparison with FTIR. The split in-plane bending vibration and the out-of-plane bending vibration due to planar CO_3_^2−^ ions appear at about 875 and 752 cm^−1^, respectively.

Although the presence of anhydrite has been proved by XRD in the samples with SF additions, too, its absorption bands are overlapped by intense silicate vibrations.

It is clearly visible from the Figure 4 that the region of silicate stretching vibrations in Q^2^ units moved to higher wavenumbers, mainly as a result of SF addition, which denotes more pronounced polymerization of derieketten chains [27,46]. ^29^Si MAS NMR studies of Bosque et al. [47] confirmed that the C-S-H gel polymerizes linearly and, therefore, the increase of only Q^1^ (an end group of silicate chain), and mainly Q^2^ units (a middle group) can be observed during this process. The reason for SF effect at any temperature is the acceleration of C_3_S hydration, as well as pozzolanic reactions, leading to the formation of other C-S-H phases [47,48]. The promotion of polymerization induced by higher temperatures is also well known and documented by many researchers [5,49]. However, by comparing the results for samples prepared under the conditions selected in this study, no shift of absorption bands originating from Q^2^ groups to higher wavenumbers with increasing temperature is recorded. It is caused by decomposition and higher condensation degree of formed C(-A)-S-H phases accompanying transformation and crystallization of primary hydration products at very high temperatures. Alonso and Fernandez [50] demonstrated that the evolution of the ^29^Si MAS-NMR spectrum of hardened cement pastes upon the heating between 100 and 200 °C (heating rate of 1 °C min^−1^) is characterized by low decrease of intensities in Q^1^ and Q^2^ tetrahedra, and the formation of some new anhydrous nesosilicate phase, type Q^0^. Cong and Kirkpatrick [1] investigated hardened C-S-H samples after the heating to 110 °C (for 2 h) and 200 °C (for 3 h). Their results indicated that the heating of C-S-H decreases the local structural order and increases polymerization of the structure. At the same time, the presence of Q^3^ sites (branching site tetrahedra) found in their ^29^Si MAS-NMR spectra was explained by breaking down Si-OH linkages on Q^2^ sites. Eventually, it can result in the formation of Si-O-Ca linkages with the interlayer Ca^2+^.

Accordingly, in the infrared spectra of S30_20, the sharp absorption peak emerges at 1199 cm^−1^, which can be assigned to the Si–O stretching vibration in Q^3^ sites [51]. This peak, indicating cross-linking of the silicate chains, is characteristic of the 9Å tobermorite structure (Ca_4_(Si_6_O_15_)(OH)_2_(H_2_O)_5_, C_4_S_6_H_6_). Other peaks, ascribed to vibrations from the structure of tobermorite, are at about 1052 cm^−1^ (ν_as_Si–O–Si), 997 cm^−1^ (νSi–O–), 977 cm^−1^ (νSi–O–), and 935 cm^−1^ (νSi–O–), which are all attributed to asymmetric stretching vibrations of SiO_4_ tetrahedra at 668 and 603 cm^−1^ (ν_s_Si–O–Si), and 518 and 473 cm^−1^, due to Si–O–Si and O–Si–O bending vibrations, respectively. Positions of these bands correspond well with those reported in [52].

A higher amount of SF resulted in another decrease in CaO/SiO_2_ molar ratio from 1.05 to 0.56, and it changed the thermodynamic stability of the system. Instead of tobermorite, calcium hydrogen silicate, gyrolite, and a smaller amount of cowlesite were demonstrated as more thermal stable phases based on XRD. The existence of Ca-OH bonds in the gyrolite structure, usually manifesting as a sharp peak at about 3634 cm^−1^ [53], is partially hidden by a wide stretching vibration band of water, and it emerges only as a shoulder. Other absorption bands are particularly assigned as follows: 1123 and 1037 cm^−1^ (ν_as_Si–O–Si from Q^3^), 784 and 676 cm^−1^ (ν_s_Si–O–Si), 613 cm^−1^ (ν_L_(OH^-^)), 596 cm^−1^ (δSi-O-Si), 478 and 467 cm^−1^ (δO-Si-O) [54,55,56].

### 3.2. XRD

As a result of the complexity of the studied systems, the analysis of the MIR spectra would be very difficult or nearly impossible without performed XRD. XRD patterns of the samples corresponding to boundary compositions and applied conditions (220 °C, 2.0 MPa) are shown in Figure 5. Irrespectively of the composition, unreacted clinker phases, C_3_S and C_2_S, are still present in the samples. The initially formed ettringite (Ca_6_Al_2_(SO_4_)_3_(OH)_12_·26H_2_O, C_6_A$\bar{S}$_3_H_32_), confirmed in the 24 h samples, decomposed to form anhydrite. Unlike the FTIR analyses, which indicated the presence of various carbonates, only calcite is confirmed by XRD. Pozzolanic reactions of SF resulted in the consumption of portlandite, which was evidenced only in the sample without its addition (S0_20). In addition, the formation of α-C_2_SH and jaffeite was prevented by SF, and these phases were determined only in the sample without it (S0_20). Whilst pure cement paste led to the formation of calciochondrite, katoite, and hibschite, calcium hydrogen silicate, cowlesite, and gyrolite were identified as crystalline phases in S50_20. Detected katoite and hibschite belong to the intermediate members of the hydrogrossular group, and both occur in the cubic form (reference number of XRD pattern for katoite: 01-077-1713 PDF-2 database, for hibschite: 01-075-1690 PDF-2 database).

### 3.3. Thermal Analysis

**Figure 6:**
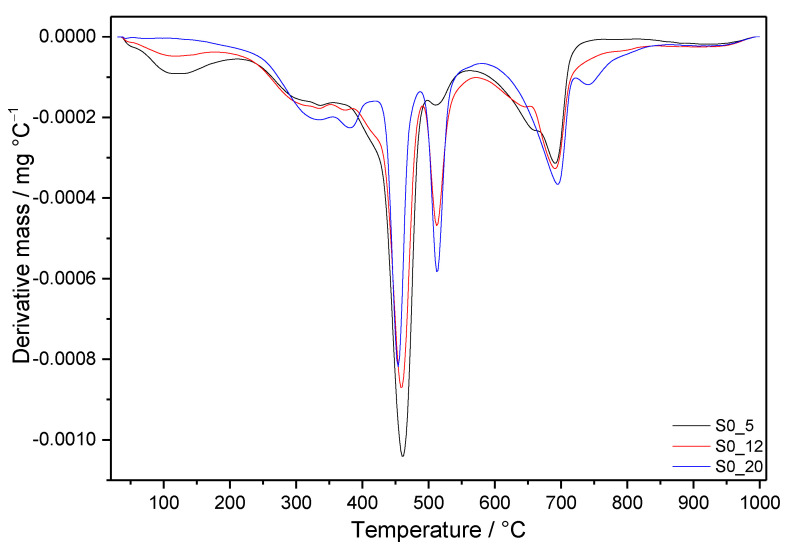
DTG curves of samples without SF addition after 7 days of hydrothermal curing.

**Figure 7:**
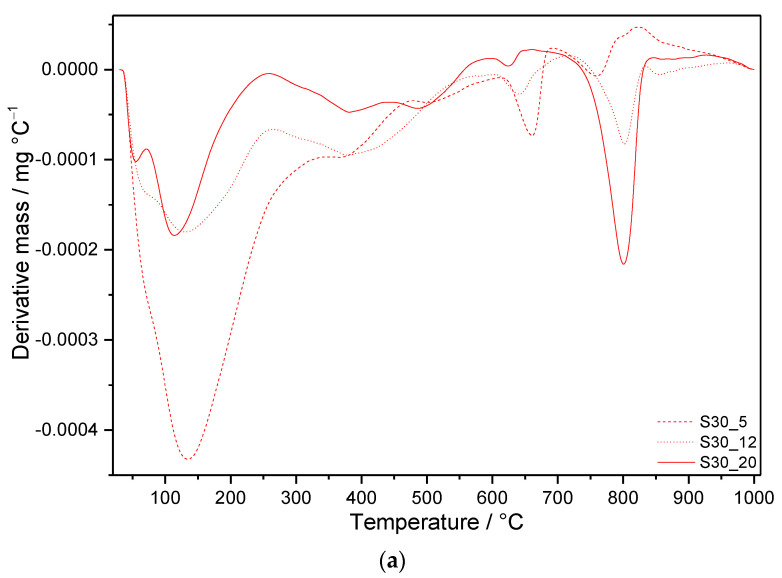

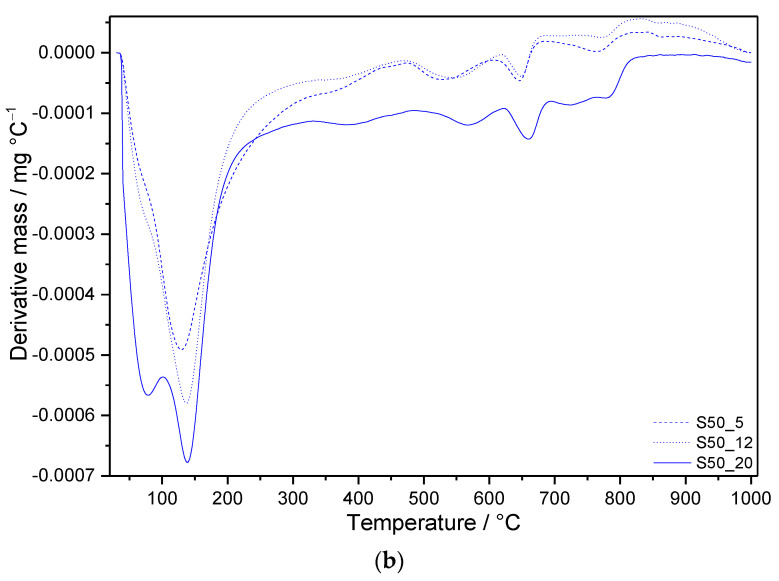
DTG curves of samples with (**a**) 30 mass% SF and (**b**) 50 mass% SF addition after 7 days of hydrothermal curing.

**Figure 8:**
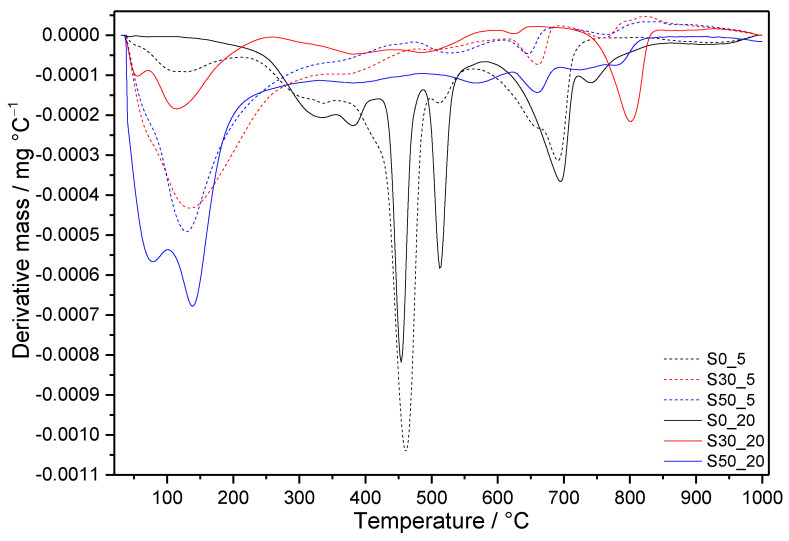
Comparison of DTG curves of samples with and without SF additions submitted to the modest and the most severe hydrothermal conditions for 7 days.

**Figure 9:**
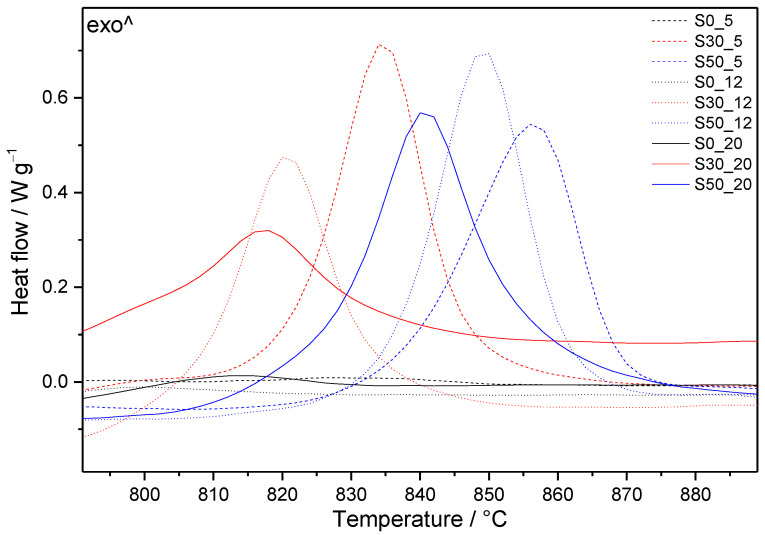
Segment of DSC curves of samples with and without SF additions submitted to different hydrothermal conditions for 7 days.

The DTG curves shown in Figure 6 and Figure 7 allow following the influence of the applied conditions on the individual cement compositions, whilst Figure 8 summarizes the effect of silica fume in the case of boundary-applied conditions. The DSC curves of all prepared samples are depicted in Figure 9 and the mass losses observed in individual temperature intervals are summarized in Table 4.

#### 3.3.1. Samples without SF Addition

The DTG and DSC curves of all prepared samples are depicted in Figure 6, Figure 7, Figure 8 and Figure 9. For a more detailed presentation, mass losses observed in individual temperature intervals are summarized in Table 4.

In case of samples without SF addition (S0), the DTG peak in the area of C-S-H decomposition temperatures decreases with increasing pressure and temperature (Figure 6). On the contrary, the intensity of DTG peaks at higher temperatures, which are attributed to the decomposition of C-A(-S)-H phases, increases with more severe conditions of hydrothermal curing. Based on previously discussed results, particular peaks with maxima at around 320 and 380 °C are connected to the decomposition of hydrogrossular phases.

The higher the temperature and pressure, the lower the mass loss between 400 and 480 °C is detected. In addition to portlandite, α-C_2_SH decomposes in the same temperature interval. Together with the decreasing intensity of this peak, its maximum moves to lower temperatures. Conversely, the DTG peak with a maximum near 510 °C, assigned to jaffeite, increases with more severe hydrothermal conditions. Comparing with the results of other analyses, it can be concluded that whilst the amount of α-C_2_SH decreases with increasing pressure and temperature, the amount of jaffeite seems to increase in this composition. Such findings match with some of our previous studies [31,32] and information from the literature [57]. Although both these phases are usually observed in the systems with CaO/SiO_2_ close to 3, they should be a subject of more detailed investigation, which would allow estimating more precise connections between these phases. For example, Blanc et al. [29] considered jaffeite to be thermodynamically stable, whilst α-C_2_SH was shown to be a metastable one. They assumed that the reduced stability field of this phase is wedged between afwillite and jaffeite, and it is probably too strongly constrained by jaffeite. However, following some observations, at some conditions, the transition with hillebrandite occurs at 159 °C. Another study confirmed the simultaneous formation of α-C_2_SH and jaffeite. However, whilst the amount of α-C_2_SH continuously increased, that of jaffeite remained almost constant with prolonging hydrothermal curing up to the observed 28 days [33].

Above 600 °C, besides the significant DTG peak with a maximum of around 690 °C, the shoulder appears at a lower temperature in case of two modest hydrothermal conditions. In addition, a new peak at about 745 °C can be observed on the curve of S0_20. In general, the following reasons could lead to the existence of various peaks in this temperature area: different modifications of calcium carbonate (calcite, aragonite, vaterite), their different origins (mainly from CH or from C-S-H), and different degree of crystallinity, carbonation during preparation, and manipulation with the samples (including the effect of solvents used to stop the hydration), but also the decomposition of some highly crystalline phases other than calcites [58].

The effect of the preparation and manipulation procedure can be eliminated, since it was kept constant. In addition, it is difficult to determine the origin of the observed carbonates. The consensus is that the carbonation of portlandite is more pronounced at the beginning of carbonation [4], whilst, e.g., according to Thiery et al. [59], C–S–H reacts continuously and at a relatively constant rate. Although no different times of hydration were studied here, the decomposition of carbonates from C-S-H is expected to occur at lower temperatures than that from portlandite. Based on the results of FTIR and XRD for S0 samples, all the present peaks in this temperature area can be assigned to the calcite only. Thus, lower decomposition temperatures of calcite than those observed in some other studies [59] probably relate to its less perfect crystalline state.

#### 3.3.2. Samples with SF Addition

Additions of SF resulted in substantially higher amounts of thermally stable products with lower C/S ratios, which did not undergo undesired transformation into α-C_2_SH and jaffeite (except for S30_12 and S30_20) (Figure 7 and Figure 8). This is clearly shown by the intensity of the first decomposition peak increasing with the amount of SF under the same curing conditions. In line with other analyses, the total depletion of portlandite by pozzolanic reactions is evidenced by its missing DTG peak. This relates also to the lower amount of detected carbonates in comparison to S0 samples (Table 4). DTG peaks appearing between 480 and 615 °C on the curves of S50 samples refer to the second mass loss from gyrolite [60]. Other DTG peaks at temperatures close to the decomposition of jaffeite suggest the formation of different modifications of calcium carbonate, as is demonstrated by MIR spectra of S30 and S50 samples submitted to more severe hydrothermal conditions. On the contrary, the DTG effects at higher temperatures belong to the well-crystallized calcite.

As a result of the lower C/S ratio in these samples, an exothermic effect appears on the DSC patterns at temperatures above 800 °C (Figure 9). It is assigned to the transformation of present phases, such as tobermorite and gyrolite, to anhydrous calcium silicate phases, e.g., wollastonite (CaSiO_3_, CS) [61,62,63]. The maximum of these effects moves to lower temperature, with increased temperature and pressure of hydrothermal curing. At the same time, the shapes of peaks change; they become lower and wider. On the contrary, higher SF additions shift the maxima of exothermal peaks to higher temperatures.

### 3.4. A Consideration of the Changes Taking Place in the Systems

Regardless of the applied conditions, ettringite, nanocrystalline C(-A)-S-H (often katoite from unstable C_4_AH_13_ and C_2_AH_8_, which are formed by rapid hydration of C_3_A), and portlandite are the first hydration products formed in the systems based on Portland clinker under standard curing conditions. The traces of calcite can be determined as well. Since the temperatures above 100 °C were selected for this study, primary hydrations products transform into more stable phases, and a new equilibrium must be attained, which depends on the initial composition and specific hydrothermal conditions. Thus, the final phase composition of cementitious material submitted to hydrothermal curing is determined by the thermodynamics of the system, whilst the quantity of the formed phases depends on the kinetics, which also relates to the applied temperature, pressure, and length of the hydrothermal curing. However, because the precipitation and the dissolution processes can be slower, thermodynamic equilibrium may not be reached, and thermodynamically stable phases may not form spontaneously [64]. This is the reason why also metastable phases can be found in the systems.

Considering our systems and samples without SF addition, besides carbonates and anhydrite, originating from the reaction of hydration products with CO_2_ and temperature decomposition of ettringite, respectively, portlandite, α-C_2_SH, jaffeite, and hydrogrossular phases, namely hibschite and katoite (with C/S = 4.6, C/A = 1.5), were confirmed regardless of the applied hydrothermal conditions. In addition, Si–O stretching vibration from C-S-H was detected in the samples submitted to lower temperatures and pressures. The higher the temperature and the pressure, the increased amount of jaffeite and hydrogrossular phases was evidenced. On the contrary, the presence of C-S-H was not further observed, and the amount of α-C_2_SH seems to be decreased. In the case of the most severe hydrothermal conditions, the presence of calciochondrodite was uniquely determined. All the formed phases reflect a high C/S ratio of the system and were observed also by other researchers.

Going to the mechanism of performing reactions and considering the initial composition of the system, clinker phases react with water to form first a C-S-H gel, which on heating converts to crystalline α-C_2_SH and jaffeite. Based on our previous results, both these phases form together and already in the first hours of hydration. By increasing the temperature and pressure of curing, α-C_2_SH as the phase with lower thermal stability can be transformed to calciochondrite with C/S = 2.5. At the same time, higher amounts of thermally more stable jaffeite are formed from the rest of the amorphous C-S-H gel. According to the received results, hydrogrossular phases are the only Al^III+^ bearing phases formed at studied conditions owing to their stability.

Contrary to the mentioned, the decrease of C/S ratio with SF prevented the described transformations of primary hydration products, and the more pronounced polymerization of silicate chains can be observed by FTIR. Both jaffeite and α-C_2_SH were evidenced only in the case of lower SF addition and more severe hydrothermal treatment. Portlandite was consumed in pozzolanic reactions of SF already when 30 mass% cement was replaced by SF, resulting in the formation of other and thermally stable C(-A)-S-H phases. Lower SF addition and higher temperatures and pressures favor the formation of the cross-linking structure of 9 Å tobermorite (with C/S = 0.7). Contrary to the stability diagrams based on thermodynamic considerations only, according to which tobermorite should precipitate even at 20 °C and be more stable than the other C-S-H phases, it has never been neoformed at ambient temperature. Its crystallization from calcium released by decalcification of the cement phases (portlandite dissolution and C-S-H decalcification) is supposed in unblended cement systems [65]. In addition, according to Houston et al. [66], who conducted batch experiments of CaO-SiO_2_-Al_2_O_3_ system in alkaline solutions at 150 °C, tobermorite formation takes place through the formation of amorphous and semi-crystalline C-S-H followed by the growth of semi-crystalline tobermorite and re-crystallization of the tobermorite solid. The indirect formation of tobermorite was supported also by other researchers in the systems containing an additional source of S^IV+^, such as SF [67,68].

A higher amount of SF resulted in another decrease in C/S ratio, and instead of tobermorite, calcium hydrogen silicate (with C/S = 0.5) and gyrolite (with C/S = 0.7) were demonstrated as more thermal stable phases. Instead of hibschite and katoite, cowlesite (with C/S = 0.3, C/A = 0.5) was formed, reflecting a decreased amount of Ca^2+^ in comparison with Al^III+^ as well as a lower C/S ratio following the quantity of SF at the expense of clinker phases. Siauciunas and Baltakys [69] demonstrated the formation of gyrolite through intermediary compounds C-S-H (I) and Z-phase. According to the findings of Taylor [70], which were slightly and more recently modified by Meller et al. [71], also the formation from tobermorite can be considered.

## 4. Conclusions

Portland cement CEM I 52.5 R was substituted up to 50 mass% of SF and submitted to three regimes of hydrothermal curing for 7 days (0.5, 1.2, and 2 MPa and 165, 195, and 220 °C, respectively). A detailed study of the formed phases was enabled by a combination of MIR, XRD, and TGA analyses, which is otherwise limited by the complexity of these systems. The obtained results can be summarized as follows.

- Calciochondrite, katoite, hibschite, α-C_2_SH, jaffeite, portlandite, anhydrite, and calcite were identified in the hydrothermally cured pure cement pastes.
- In case of two modest hydrothermal regimes, poorly crystalline C-S-H was still present in the samples without SF addition.
- Whilst the amount of α-C_2_SH decreased, the amount of jaffeite increased with increasing temperature and pressure.
- The transformation of primary hydration products to α-C_2_SH and jaffeite was pre-vented by SF additions. In case of its lower substitution level (30 mass%), only the traces of jaffeite were revealed by the performed analyses. Higher substitution led to the total suppression of the formation of these phases.
- Portlandite was already depleted by the lower replacement of cement, which was related to the lower carbonation degree in particular samples. In addition to calcite, other modifications of calcium carbonate were identified at higher temperatures and pressures.
- 30 mass% SF resulted in the formation of stable tobermorite, whilst calcium hydrogen silicate, gyrolite, and smaller amounts of cowlesite were identified as the main crystalline phases in the samples with 50 mass% SF.
- The main advantage of the implementation of the FTIR technique, in comparison to the other ones, lies in the possibility to follow the changes in the silicate hydrates structure. Especially in the region of silicate stretching vibrations, their shifts can clearly denote polymerization of the chains or decomposition and higher condensation degree of the formed phases accompanying transformation and crystallization of primary hydration products at very high temperatures.
- In the region of higher wavenumbers, the replacement of [SiO_4_]^4−^ tetrahedra by 4OH^−^, for example, the formation of different hydrogrossular phases, can be observed.

## Figures and Tables

**Table 1:** Composition of prepared samples (in mass%) and their designation according to the applied hydrothermal conditions.

Sample	Applied Conditions	*w*_pc_	*w*_SF_
S0_5	0.5 MPa, 165 °C	100	-
S0_12	1.2 MPa, 195 °C		
S0_20	2.0 MPa, 220 °C		
S30_5	0.5 MPa, 165 °C	70	30
S30_12	1.2 MPa, 195 °C		
S30_20	2.0 MPa, 220 °C		
S50_5	0.5 MPa, 165 °C	50	50
S50_12	1.2 MPa, 195 °C		
S50_20	2.0 MPa, 220 °C		

**Table 2:** Oxide composition and specific surface of the used Portland cement and SF. Depicted values of a specific surface area represent the average of three measurements.

	Oxide Composition (Mass%)						Specific Surface (m^2^ kg^−1^)	
	CaO	SiO_2_	Al_2_O_3_	Fe_2_O_3_	MgO	SO_3_		
CEM I 52.5 R	61.84	21.84	5.15	2.85	1.56	3.33	Blaine	560.9 ± 0.8
SF	0.50	97.10	0.21	-	0.40	-	BET	15,000

**Table 3:** Mineral composition of CEM I 52.5 R given by producer.

Type of Used Cement	Mineral Composition (Mass%)						
	C_3_S	C_2_S	C_3_A (ort.)	C_3_A (cub.)	C_4_AF	Free Lime	MgO
CEM I 52.5 R	60.36	11.38	5.72	2.32	8.35	2.69	0.24

**Table 4:** Total mass loss, and mass loss of prepared samples in different temperature intervals corresponding to particular hydration products. Displayed temperature intervals are only approximate. Corresponding mass losses were determined according to the particular DTG curves.

Sample	S0_5	S30_5	S50_5
Temperature Interval (°C)	Mass Loss (Mass%)		
	165 °C, 0.5 MPa		
r.t.–400 (C(-A)-S-H, C-A-H)	5.7	15.0	12.9
400–480 (CH, α-C_2_SH)	5.4		
490–560 (jaffeite)	1.1		
560–1000 (carbonates)	4.4	1.8	1.2
Total mass loss (%)	16.6	16.8	14.1
**Sample**	**S0_12**	**S30_12**	**S50_12**
	**195 °C, 1.2 MPa**		
r.t.–400 (C(-A)-S-H, C-A-H)	4.3	10.3	13.8
400–480 (CH, α-C_2_SH)	4.4		
490–560 (jaffeite)	2.0		
560–1000 (carbonates)	4.5	2.3	1.4
Total mass loss (%)	15.2	12.6	15.2
**Sample**	**S0_20**	**S30_20**	**S50_20**
	**220 °C, 2.0 MPa**		
r.t.–400 (C(-A)-S-H, C-A-H)	4.3	8.0	12.8
400–480 (CH, α-C_2_SH)	2.6		
490–560 (jaffeite)	2.1	1.9	
490–1000 (carbonates)	4.4	2.8	1.8
Total mass loss (%)	13.4	12.7	14.6

## Data Availability

Data is contained within the article.

## Abbreviations

A	Al_2_O_3_
C	CaO
C-A-H	Calcium Aluminate Hydrate
C-A-S-H	Calcium Alumina Silicate Hydrate
C-S-H	Calcium Silicate Hydrate
$\bar{C}$	CO_2_
F	Fe_2_O_3_
FTIR	Fourier Transform Infrared Spectroscopy
H	H_2_O
M	MgO
S	SiO_2_
SF	Silica Fume
$\bar{S}$	SO_3_
TGA	Thermogravimetric Analysis
XRD	X-Ray Diffraction
